# Hepatic fascioliasis presenting with bile duct obstruction: a case report

**DOI:** 10.11604/pamj.2017.28.44.11532

**Published:** 2017-09-18

**Authors:** Rachid Lefryekh, Ahmed Bensaad, Fatimazahra Bensardi, Khalid Elhattabi, Mounir Bouali, Bessam Daif, Abdelaziz Fadil, Zakaria Jaouhari, Tazi Hicham, Aziz Hamdani, Maha Soussi Abdalaoui

**Affiliations:** 1 Surgical Emergency Unit P35, University Teaching Hospital Ibn Rochd, Casablanca, Morocco; 2 Department of Parasitology, University Teaching Hospital Ibn Rochd, Casablanca, Morocco

**Keywords:** Hepatic Fascioliasis, Bile ducts, obstructive jaundice

## Abstract

Fascioliasis is a zoonotic infection caused by a liver trematode: fasciola hepatica; which commonly affects cattle and sheep, humans are accidental hosts. Several cases have been reported in the literature worldwide with a large geographical distribution. We present a case of bile duct obstruction due to a hepatic fascioliasis, successfully treated with both a combined surgical and medical approaches. A high index of suspicion should be kept in mind for all cases of obstructive jaundice, especially in areas in which human fascioliasis infection is repeatedly reported.

## Introduction

Biliary fascioliasis is a rare infection of the hepato-biliary system, caused by the trematode Fasciola hepatica. There has been recently a well recognized worldwide risk of fasciola hepatica infection, and it is becoming an important public health problem, with an estimated 2,5 million people infected in about 61 countries [1]. Few cases have been reported in Morocco, in which it is thought that many human cases of fasciola hepatica infections are been underdiagnosed. We report a case of biliary fascioliasis in its chronique phase, diagnosed on magnetic resonance imaging (MRI) as a hilar cholangiocarcinoma, for which surgery was indicated, as the biliary sepsis could not be controlled.

## Patient and observation

**Figure 1:**
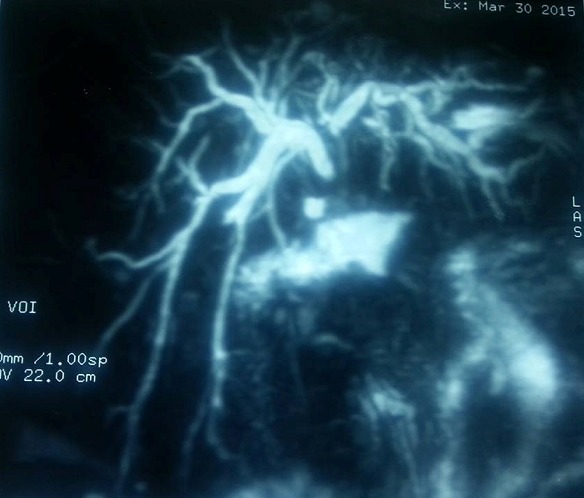
Magnetic retrograde cholangiopan¬creatography (MRCP) revealing dilatation of the intrahepatic bile ducts with filling defect of the upper-portion of the common bile duct

**Figure 2:**
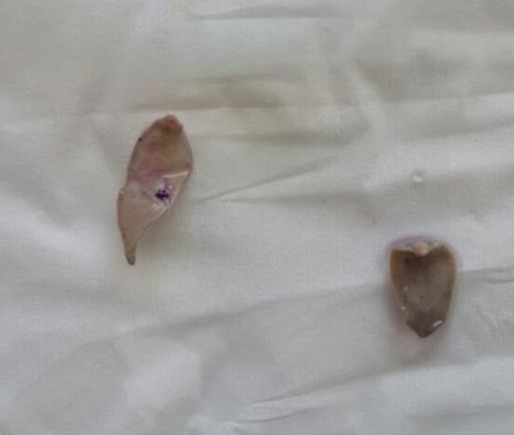
Fasciola hepatica adult worm

A 40-year-old woman who had a right upper quadrant (RUQ) pain and discomfort for several months was referred to our general surgery department with a suspected cholangiocarcinoma diagnosis. Her main complaint was a mild jaundice, fever, and pain in the RUQ of the abdomen. She had a history of cholelithiasis treated uneventfully with a laparoscopic cholecystectomy. No upper gastrointestinal bleeding, hematochezia, facial rash, itching, or generalized pruritus were present. Abdominal ultrasound revealed mild intra-hepatic biliary dilatation, with bile wall thickening, and no stones were observed. Blood tests revealed no eosinophilia (350 /microliter), while asparate aminotransferase and alanine aminotransferase values were two times the upper limit, 68IU/l and 100IU/L respectively and; Gamma glutamyl-transferase was six times greater (300IU/l). Total bilirubin and direct bilirubin levels were ten and seven times the upper limit, 106mg/l and 80mg/l respectively. Tumor markers were within the normal range. (CA 19.9: 30U/ml; CEA: 3.9U/ml) Since no definite diagnosis could be reached on ultrasonography, magnetic resonance imaging (MRI) was used and a diagnosis of hilar cholangiocarcinoma was suspected (Figure 1). Given the uncontrolled sepsis developed by our patient, surgery was indicated and an intraoperative diagnosis of hepatic fascioliasis was made (Figure 2). The biliary tree was evacuated from mobile parasites instrumentally and the CBD was sutured over a T-tube. Triclabendazole 10mg/Kg/day was successfully used on a single dose regimen. Final cholangiogram realized on the Post-operative day 15 showed no filling defects, and the T-tube was removed. The patient has remained asymptomatic for 4 months.

## Discussion

Fascioliasis is a zoonotic infection caused by a trematode of the liver, fasciola hepatica. Adult large flukes of Fascioliasis are flat worms; measuring up to 2.0 to 4.0 cm long, 1 to 1.5 cm wide. Humans are considered accidental hosts and the infection cycle starts with the ingestion of contaminated water or vegetales containing the larvae [2]. Metarcercariae excyst in the host's duodenum; and due to its invasive nature, it perforates the peritoneum and Glisson's capsule, reaching the biliary ducts through the liver parenchyma. The disease occurs in two phases: the hepatic phase, lasting 3 to 5 months, during which the young fluke penetrates the liver, symptoms of this phase include prolonged fever, hepatomegaly causing right upper quadrant abdominal pain and eosinophilia. Other non specific manifestations are anorexia, weight loss, urticaria and lymphadenopathies. The biliary or chronic phase, represented by the migration from venous radicals into the biliary tree, begins after 6 months. Half of the patients are asymptomatic, and very few cases develop features of biliary tree obstruction, as those presented in this case [2, 3]. Ultrasonographic (USG) findings are generally not specific, and may show heterogeneity of the liver parenchyma and some hypoechoic nodules in the acute phase. During the biliary phase, USG findings are those of biliary obstruction, mainly biliary dilatation and in some cases bile wall thickening. Although other imaging possibilities, including computed tomography (CT) and MRI have both equally good diagnosis value, CT is a more appropriate diagnostic tool, identifying the classic “tunnels and caves sign” which correspond to the path of migration of the F. hepatica from the Glisson capsule deep into the hepatic parenchyma [1, 4, 5]. Final diagnosis can be made with microscopic identification of the ova in the stool and the exact diag¬nosis can be made with the serological tests [6]. Comparatively to other case reports of human fascioliasis in the literature, cases that were diagnosed intra-operatively are very rare, and this case shows that a surgical approach can be necessary in some complicated situations. More often, the biliary phase of the fascioliasis is at best managed, when the diagnosis is made before surgery, with ERCP and sphincterotomy [1]. A single dose of Triclabendazole (TCBZ), 10-20 mg/kg/day, is actually the recommended treatment for fascioliasis, some authors consider the response to this treatment as a criterion for the diagnosis to be made. While this therapy is enough on a single dose regimen, multiple doses may be required in some patients with persistent infection [7].

## Conclusion

A high index of suspicion is necessary for all cases of obstructive jaundice, especially in areas in which human fascioliasis infection is repeatedly reported.

## Competing interests

The authors declare no competing interest.
